# Rule-derived preoperative inflammatory-lymphocyte recovery phenotype and pathological response in resected osteosarcoma: a retrospective cohort study

**DOI:** 10.3389/fimmu.2026.1925540

**Published:** 2026-09-07

**Authors:** Yang Liu, Hua Huang, Ke Yang, Daxin Gao, Tian Hao, Haiyu Zhou, Yonghong Huang

**Affiliations:** 1 The Second Hospital & Clinical Medical School, Lanzhou University, Lanzhou, Gansu, China; 2 Department of Orthopedics, Chengdu Xinhua Hospital Affiliated to North Sichuan Medical College, Chengdu, Sichuan, China; 3 Department of Orthopedics, Neijiang Hospital of Traditional Chinese Medicine, Neijiang, Sichuan, China

**Keywords:** lymphocyte recovery, neoadjuvant chemotherapy, osteosarcoma, pathological response, peripheral blood biomarkers, systemic inflammation

## Abstract

**Background:**

Pathological response after neoadjuvant chemotherapy is a key treatment milestone in osteosarcoma. We evaluated whether a rule-derived preoperative peripheral inflammatory-lymphocyte recovery phenotype constructed from routinely collected laboratory measurements was associated with poor pathological response.

**Methods:**

This single-center retrospective cohort included patients with osteosarcoma who completed neoadjuvant chemotherapy, underwent definitive surgery, had pathological response assessed, and had valid baseline (T0) and preoperative (T3) laboratory measurements. A failure score combined standardized myeloid-inflammatory burden and lymphocyte-nutritional reserve. Patients were classified as favorable recovery, intermediate recovery phenotype, or persistent recovery failure using the primary T0-T3 rule. The primary outcome was tumor necrosis <90%. The primary association was estimated using parsimonious Firth logistic regression, with modified-Poisson risk ratios and standardized absolute risks. Model comparisons used nested full-pipeline repeated cross-validation. Event-related analyses were exploratory.

**Results:**

Among 162 patients, 88 (54.3%) had poor pathological response; 65 were classified as favorable recovery, 45 as intermediate, and 52 as persistent recovery failure. Persistent failure was associated with poor pathological response versus favorable recovery (Firth OR, 5.72; 95% CI, 2.42-13.53; P<0.001; adjusted RR, 2.10; 95% CI, 1.46-3.01). Standardized risks were 37.2% for favorable recovery and 75.7% for persistent failure, corresponding to a risk difference of 38.6 percentage points (95% CI, 19.7-52.7). In nested full-pipeline cross-validation, the phenotype model had an AUC of 0.641 and did not outperform the continuous T3 score (AUC, 0.665); its out-of-fold calibration slope was 0.587 (95% CI, 0.220-0.953). Exploratory associations were observed for event-free survival (EFS) and pulmonary metastasis, whereas overall survival (OS) was immature with 24 deaths.

**Conclusion:**

A rule-derived preoperative peripheral inflammatory-lymphocyte recovery phenotype was associated with pathological response among patients who completed neoadjuvant chemotherapy and underwent definitive surgery for osteosarcoma. The association was robust across alternative definitions and sensitivity analyses; however, the categorical phenotype did not outperform the continuous preoperative T3 score, and out-of-fold calibration indicated residual overfitting. Event-related findings and biological interpretation require external validation.

## 1 Introduction

Osteosarcoma is the most common primary malignant bone tumor in children, adolescents, and young adults, and cure for localized high-grade disease still depends on multi-agent chemotherapy combined with complete surgical resection (1–3). The modern treatment backbone improved survival compared with surgery alone, but progress has been modest over recent decades, particularly for patients with metastatic, recurrent, or chemotherapy-resistant disease (4–6).

Histological response after neoadjuvant chemotherapy remains one of the most informative treatment milestones in osteosarcoma. Large cooperative-group analyses have shown that tumor site, size, metastatic status, surgical remission, and response to preoperative chemotherapy are major prognostic factors (4). The commonly used threshold of at least 90% tumor necrosis identifies patients with favorable histological response, whereas necrosis below this threshold defines a poor response group with higher relapse risk (4–6). However, pathological response is only available after resection, so it does not fully address the need for earlier markers of treatment-period vulnerability.

The immunological context of osteosarcoma provides one reason why host response during chemotherapy may be clinically relevant. Osteosarcoma contains diverse immune, stromal, and myeloid compartments, and recent single-cell and tumor microenvironment studies have emphasized inter-patient heterogeneity in immune-cell states and intercellular communication (7–10). Clinical trials of immune checkpoint blockade have produced limited activity in unselected bone sarcoma cohorts, suggesting that the immunological barriers to effective tumor control are not adequately captured by tumor histology or stage alone (11–13).

Peripheral blood biomarkers offer a practical window into systemic inflammation and immune reserve. In osteosarcoma, baseline neutrophil-to-lymphocyte ratio, platelet-to-lymphocyte ratio, systemic immune-inflammation index, prognostic nutritional index, and related markers have been associated with prognosis in several retrospective cohorts and meta-analyses (14–18). Yet most available work relies on pretreatment or preoperative single time points. A dynamic NLR study in osteosarcoma showed that changes during treatment can add prognostic information beyond baseline values, supporting the broader idea that treatment-period immune-inflammatory adaptation may be informative (19).

The immune-resilience framework describes the capacity to preserve or restore favorable immune function while limiting harmful inflammation under biological stress (20). The routine laboratory indices available in this cohort cannot establish that construct directly; instead, they provide an observable summary of peripheral inflammation, lymphocyte status, and nutritional reserve during treatment. We therefore evaluated a rule-derived T0-T3 preoperative peripheral inflammatory-lymphocyte recovery phenotype and tested its association with pathological response. Comparisons with simpler biomarkers and event-related outcomes were prespecified as secondary or exploratory analyses.

## 2 Methods

### 2.1 Study design and cohort

This single-center retrospective cohort study identified patients with osteosarcoma diagnosed between February 2014 and June 2025 and followed through June 30, 2026. Patients received neoadjuvant chemotherapy followed by definitive surgery, and all records were analyzed after de-identification. Reporting followed the STROBE framework and REMARK principles where applicable (21, 22). The institutional ethics committee approved the study (approval no. lzshmed2500152) and waived written informed consent because de-identified retrospective clinical data were used.

**Figure 1:**
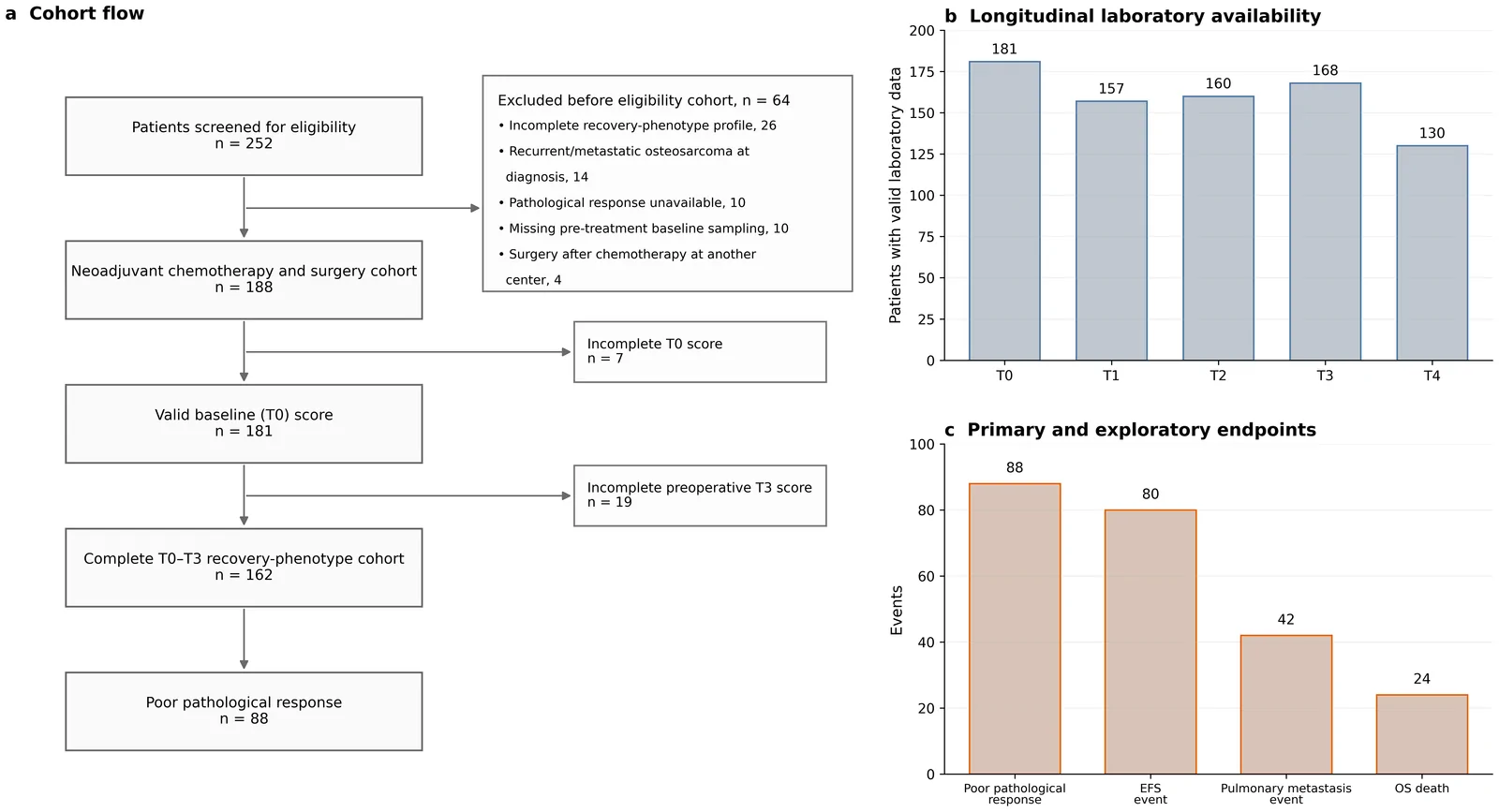
Cohort construction, laboratory availability, and endpoint counts. **(A)** Flow from screened records to the primary recovery-phenotype cohort; exclusion branches show records excluded before cohort eligibility and patients without complete T0 or preoperative T3 scores. **(B)** Availability of valid laboratory data across treatment windows. T0 and T3 were required for classification; T1, T2, and T4 were descriptive or secondary. **(C)** Counts of poor pathological response and exploratory event-related outcomes. EFS included recurrence, progression, pulmonary metastasis, or death. Pulmonary-metastasis analyses were restricted to patients without metastasis at diagnosis.

The primary analytic population was restricted to patients who completed neoadjuvant chemotherapy, underwent definitive surgery, had pathological response assessed, and had valid T0 and T3 measurements for every component required to calculate the primary score. Patients without surgery, without a definitive pathological specimen, or without complete T0/T3 score components were not assigned a recovery phenotype. Cohort construction, laboratory availability, and endpoint counts are shown in **Figure 1**; **Supplementary Figure 1**.

### 2.2 Laboratory windows, timing, and contamination handling

T0 represented the last valid assessment before neoadjuvant chemotherapy; T1 and T2 represented early and mid-treatment assessments; T3 represented the last valid post-neoadjuvant, preoperative assessment; and T4 represented postoperative or pre-adjuvant assessment. T0 and T3 were required for phenotype classification. T1, T2, and T4 were used only for descriptive longitudinal displays or secondary analyses. T3 occurred a median of 101 days (interquartile range [IQR], 90-113) after the first chemotherapy administration and 3 days (IQR, 2-5) before surgery; accordingly, the phenotype was evaluated as preoperative risk stratification rather than an early-treatment predictor.

Laboratory records were flagged when obtained in prespecified windows potentially affected by infection or fever, antibiotics, granulocyte colony-stimulating factor, systemic corticosteroids, transfusion, albumin infusion, or other invalid conditions. These indicators were examined in adjusted and exclusion sensitivity analyses. The score required complete components at a given time point; partial-domain averaging and outcome-based imputation were not used.

### 2.3 Construction of the peripheral inflammatory-lymphocyte failure score

At each valid time point, NLR was neutrophils/lymphocytes; SII was platelets x neutrophils/lymphocytes; SIRI was neutrophils x monocytes/lymphocytes; CAR was C-reactive protein/albumin; and PNI was albumin + 5 x lymphocyte count. NLR, SII, SIRI, and CAR were natural-log transformed. Each component was standardized as z=(value-T0 reference mean)/T0 reference standard deviation using the 181 patients with a valid baseline value. The myeloid-inflammatory score was the unweighted mean of z-ln(NLR), z-ln(SII), z-ln(SIRI), and z-ln(CAR). The lymphocyte-nutritional reserve score was the unweighted mean of z-lymphocyte count, z-albumin, and z-PNI. The failure score was myeloid-inflammatory score minus reserve score, so higher values indicated a less favorable peripheral profile (**Supplementary Table 1**).

The components are not biologically or algebraically independent: NLR, SII, and SIRI reuse lymphocyte count, while PNI deterministically incorporates albumin and lymphocyte count. The score was therefore treated as a transparent composite summary rather than a set of independent immune domains. Correlation, variance-inflation, component-contribution, leave-one-component-out, leave-one-domain-out, and nonredundant primitive-variable analyses were performed to quantify this overlap (**Supplementary Tables S3, S4**; **Supplementary Figures 2, S3**).

### 2.4 Primary rule-derived recovery phenotype

Patients were classified using the primary T0-T3 rule. Favorable recovery required a T3 failure score below the cohort median and either a T3-T0 failure-score decrease of at least 0.25 SD or failure scores below the lower-tertile cut points at both T0 and T3. Persistent recovery failure required a T3 failure score at or above the upper-tertile cut point and a T3-T0 reserve-score change <0.05. All remaining patients were classified as having an intermediate recovery phenotype. Numeric cut points were: T3 median failure score, 0.702126; T3 upper tertile, 2.067278; T0 lower tertile, -0.340821; and T3 lower tertile, -0.844179 (**Supplementary Table 2**).

The 0.05 reserve-change boundary was selected as a transparent interior operational convention within the interval compatible with the rounded patient-level assignment indicator available in the analysis dataset (>0.037 to ≤0.057). It was not optimized using pathological-response or event-related outcomes and was not considered a biologically established threshold. The primary rule reproduced all 162 patient-level assignments. A protocol-expanded rule, a T3-only rule, threshold-bootstrap stability, and full-pipeline bootstrap reclassification were evaluated as sensitivity analyses (**Supplementary Tables 11 and S12**).

### 2.5 Outcomes

The primary outcome was poor pathological response, defined as <90% tumor necrosis in the definitive surgical specimen. Robustness was examined using ordered Huvos grade, continuous necrosis percentage, median regression, and a complete-case <80% necrosis threshold. The four records without an exact necrosis percentage remained missing in percentage-based analyses and were not assigned to the non-event category.

All event-related outcomes were exploratory. EFS was measured from surgery to the first recurrence, progression, pulmonary metastasis, death, or last follow-up. Pulmonary-metastasis analyses were restricted to patients without metastasis at diagnosis; cause-specific Cox models treated death before pulmonary metastasis as a competing event, Aalen-Johansen curves estimated cumulative incidence, and Fine-Gray models estimated subdistribution hazards. OS was measured from surgery to death or last follow-up. Because only 24 deaths occurred, OS analyses were descriptive and unadjusted.

### 2.6 Covariates

The parsimonious primary model included recovery phenotype, age, sex, baseline metastasis, tumor size, and neoadjuvant regimen. The fully adjusted sensitivity model additionally included baseline lactate dehydrogenase (LDH), baseline alkaline phosphatase (ALP), number of neoadjuvant cycles, dose delay, and any treatment-period contamination. Further analyses examined baseline failure score, treatment exposures, timing, alternative population restrictions, and complete-case inclusion weighting. Covariates were selected for clinical relevance rather than stepwise significance.

### 2.7 Statistical analysis

Continuous variables are summarized as median (IQR) and categorical variables as number (percentage). Baseline and longitudinal group comparisons are descriptive because several displayed variables contribute directly to the classification rule. Kruskal-Wallis tests and chi-square tests with Monte Carlo-estimated P values when expected cell counts were sparse were used for descriptive comparisons. **Table 1** P values refer to between-group differences in T3-T0 change and are not interpreted as independent validation.

**Table 1:** Dynamic laboratory profiles from T0 to T3 by recovery phenotype.

Marker	Time	Overall	Favorable recovery	Intermediate recovery	Persistent failure	P value
NLR	T0	2.34 (1.98–2.75)	2.32 (2.00–2.89)	2.36 (2.14–2.73)	2.30 (1.92–2.63)	
NLR	T3	2.54 (1.71–4.09)	1.68 (1.29–2.09)	2.71 (2.06–3.08)	4.46 (3.43–6.31)	
NLR	Change T3–T0	0.14 (-0.64–1.77)	-0.64 (-1.17–0.25)	0.02 (-0.45–0.87)	2.32 (1.26–4.05)	<0.001
SII	T0	650.20 (510.12–763.77)	669.80 (542.20–763.70)	639.70 (496.10–768.90)	644.35 (496.43–761.40)	
SII	T3	780.10 (458.08–1241.62)	462.50 (317.00–632.80)	823.90 (583.20–1088.20)	1471.00 (1098.30–2003.05)	
SII	Change T3–T0	104.10 (-216.75–650.75)	-208.20 (-349.70–18.90)	84.70 (-135.90–467.60)	806.30 (424.75–1310.65)	<0.001
SIRI	T0	1.00 (0.82–1.22)	0.97 (0.74–1.14)	0.94 (0.83–1.17)	1.09 (0.85–1.30)	
SIRI	T3	1.14 (0.63–2.04)	0.62 (0.47–0.87)	1.31 (1.04–1.76)	2.50 (1.53–3.63)	
SIRI	Change T3–T0	0.11 (-0.30–0.91)	-0.29 (-0.64–0.03)	0.43 (-0.10–0.85)	1.44 (0.34–2.50)	<0.001
CAR	T0	0.13 (0.08–0.20)	0.12 (0.08–0.18)	0.13 (0.09–0.18)	0.15 (0.09–0.21)	
CAR	T3	0.12 (0.06–0.23)	0.08 (0.04–0.12)	0.11 (0.07–0.22)	0.21 (0.13–0.49)	
CAR	Change T3–T0	-0.01 (-0.09–0.11)	-0.05 (-0.12–0.00)	-0.01 (-0.09–0.11)	0.07 (-0.07–0.29)	<0.001
PNI	T0	50.25 (48.52–51.88)	50.30 (48.00–51.80)	50.60 (48.60–52.80)	49.65 (48.27–51.40)	
PNI	T3	48.40 (46.02–52.20)	52.80 (49.30–55.70)	48.40 (46.80–50.40)	44.90 (42.68–46.52)	
PNI	Change T3–T0	-1.55 (-4.58–2.17)	2.30 (-1.00–5.00)	-2.40 (-4.30–0.70)	-4.85 (-7.17–3.58)	<0.001
Lymphocyte count, 10^9/L	T0	1.59 (1.41–1.79)	1.60 (1.41–1.79)	1.62 (1.42–1.85)	1.58 (1.41–1.73)	
Lymphocyte count, 10^9/L	T3	1.42 (1.09–1.83)	1.80 (1.43–2.17)	1.41 (1.18–1.74)	1.08 (0.98–1.38)	
Lymphocyte count, 10^9/L	Change T3–T0	-0.19 (-0.51–0.23)	0.16 (-0.26–0.61)	-0.22 (-0.46–0.19)	-0.48 (-0.77–0.17)	<0.001
Albumin, g/L	T0	42.10 (40.70–43.60)	42.20 (40.60–43.50)	42.40 (40.70–44.00)	41.60 (40.95–43.60)	
Albumin, g/L	T3	41.30 (39.30–43.48)	43.10 (41.60–45.80)	41.40 (39.60–42.80)	39.20 (36.85–40.45)	
Albumin, g/L	Change T3–T0	-1.20 (-3.10–1.20)	1.00 (-1.60–3.80)	-1.40 (-3.40–0.90)	-3.10 (-4.62–1.30)	<0.001
Peripheral inflammatory-lymphocyte failure score	T0	-0.05 (-0.79–0.69)	-0.11 (-0.89–0.73)	-0.18 (-0.81–0.43)	0.11 (-0.51–0.77)	
Peripheral inflammatory-lymphocyte failure score	T3	0.70 (-1.13–2.84)	-1.62 (-2.80–1.02)	0.98 (0.37–1.58)	3.87 (2.84–4.71)	
Peripheral inflammatory-lymphocyte failure score	Change T3–T0	0.64 (-1.24–2.51)	-1.76 (-3.09–0.67)	1.10 (0.39–1.39)	3.75 (2.62–5.08)	<0.001
Lymphocyte-nutritional reserve score	T0	-0.01 (-0.58–0.51)	-0.04 (-0.61–0.46)	0.06 (-0.47–0.76)	-0.23 (-0.65–0.36)	
Lymphocyte-nutritional reserve score	T3	-0.64 (-1.32–0.64)	0.73 (-0.33–1.59)	-0.67 (-1.06–0.04)	-1.69 (-2.32–1.17)	
Lymphocyte-nutritional reserve score	Change T3–T0	-0.53 (-1.42–0.64)	0.73 (-0.30–1.50)	-0.62 (-1.34–0.12)	-1.57 (-2.28–1.09)	<0.001

T0 denotes baseline before neoadjuvant chemotherapy; T3 denotes the post-neoadjuvant preoperative assessment. P values are descriptive between-group tests of T3-T0 change. Because displayed variables contribute to classification, these comparisons describe the rule and are not independent validation. NLR, neutrophil-to-lymphocyte ratio; SII, systemic immune-inflammation index; SIRI, systemic inflammation response index; CAR, C-reactive protein-to-albumin ratio; PNI, prognostic nutritional index.

The primary association was estimated using parsimonious Firth penalized logistic regression. Modified-Poisson regression with robust variance provided adjusted risk ratios, and Firth-model standardization provided group-specific risks and risk differences. Fully adjusted Firth models, baseline-score adjustment, nonredundant primitive-variable scoring, alternative phenotype definitions, and pathological-outcome forms were sensitivity analyses. A 1,000-replicate patient-level full-pipeline bootstrap re-estimated T0 standardization, scores, distributional thresholds, group assignment, and effect estimates in each replicate.

Exploratory EFS and pulmonary-metastasis analyses used parsimonious Cox models adjusted for age, sex, tumor size, and regimen, with baseline metastasis additionally included for EFS. Proportional-hazards diagnostics used Schoenfeld residuals from the reported parsimonious models. Time-specific and fixed-horizon analyses assessed attenuation over follow-up. Pulmonary metastasis was additionally analyzed using Fine-Gray regression with death before pulmonary metastasis as the competing event. OS was reported using an unadjusted Cox model only. Cox-model effects are reported as hazard ratios (HRs), which quantify the instantaneous event hazard among individuals remaining at risk; Fine-Gray effects are reported as subdistribution hazard ratios (sHRs), which quantify covariate associations with the cumulative incidence function in the presence of competing death.

Model comparison used 10 repeats of five-fold nested full-pipeline cross-validation. Within each outer training fold, T0 standardization parameters, scores, distributional cut points, phenotype assignments, preprocessing, and ridge-penalty selection were re-estimated before prediction in the held-out fold. Models included clinical variables alone, clinical variables plus recovery phenotype, continuous T3 score, T0 score plus change, or simple marker changes. Performance was summarized by AUC, Brier score, out-of-fold calibration intercept and slope, and repeat-specific ranges. Paired immune-subset data were analyzed using log-scale baseline-adjusted ANCOVA with Holm correction; availability-related selection was examined using standardized mean differences. Phenotype-by-regimen and phenotype-by-treatment-era interactions were tested formally. Analyses were performed in Python 3 using the package versions reported in **Supplementary Code S1** and the accompanying environment file. Two-sided P<0.05 was used without implying confirmatory significance for secondary or exploratory analyses.

The categorical phenotype was benchmarked against absolute lymphocyte recovery, changes in NLR, albumin, CRP, and CAR, a combined simple-marker model, and the continuous T3 score. Incremental likelihood-ratio tests evaluated whether T1/T2-derived longitudinal slope added information beyond the continuous T3 score. Time-stratified training/test splits were used only as internal calendar-time analyses; they were not considered external validation.

Because immune-subset measurements were available in only 24 paired patients, those analyses were explicitly exploratory. Estimates were reported as baseline-adjusted geometric mean ratios with 95% confidence intervals, with Holm-adjusted P values across all subset tests. Regimen-specific response rates and phenotype distributions were reported before testing the global phenotype-by-regimen interaction.

## 3 Results

### 3.1 Cohort construction, timing, and baseline characteristics

Among 188 screened patients with core clinical information, 162 had complete T0 and T3 score components, underwent definitive surgery, and had pathological response assessed. Poor pathological response occurred in 88 patients (54.3%). The exploratory EFS definition identified 80 events, and 24 deaths occurred. Pulmonary-metastasis analyses included 147 patients without metastasis at diagnosis, with 42 pulmonary metastases and 15 deaths before pulmonary metastasis (**Figure 1**; **Table 2**).

**Table 2:** Exploratory event-related outcomes by recovery phenotype.

Outcome and model	N	Events	Effect	P value
EFS: persistent failure vs favorable recovery	162	80	HR 4.86 (2.72-8.65)	<0.001
Pulmonary metastasis cause-specific hazard	147	42	HR 6.81 (2.87-16.14)	<0.001
Pulmonary metastasis subdistribution hazard	147	42	sHR 6.50 (2.76-15.30)	<0.001
OS: unadjusted, immature endpoint	162	24	HR 1.28 (0.49-3.33)	0.612

All event-related analyses are exploratory. EFS and pulmonary-metastasis cause-specific models were parsimoniously adjusted for age, sex, tumor size, and regimen; baseline metastasis was additionally included for EFS. Fine-Gray analysis treated death before pulmonary metastasis as a competing event. Pulmonary-metastasis analyses were restricted to patients without metastasis at diagnosis. OS was unadjusted because only 24 deaths occurred. Favorable recovery was the reference.

The recovery phenotype comprised 65 patients with favorable recovery, 45 with an intermediate recovery phenotype, and 52 with persistent recovery failure. Median age was 19.0 years (IQR, 15.2-25.0), median tumor size was 8.6 cm (IQR, 6.4-10.4), and 15 patients (9.3%) had metastasis at diagnosis. Most tumors were extremity tumors, and the most common neoadjuvant regimens were AP and MAP (**Table 3**).

**Table 3:** Baseline characteristics by preoperative peripheral inflammatory-lymphocyte recovery phenotype.

Variable	Level	Overall	Favorable recovery	Intermediate recovery	Persistent failure	P value
Age, years		19.0 (15.2–25.0)	18.0 (16.0–26.0)	18.0 (15.0–22.0)	20.0 (16.0–29.0)	0.237
BMI, kg/m^2^		21.7 (19.5–23.6)	21.8 (20.1–23.6)	20.8 (18.5–24.1)	22.1 (19.7–23.3)	0.471
Tumor size, cm		8.6 (6.4–10.4)	8.2 (6.0–10.4)	8.3 (6.3–10.0)	9.1 (7.0–11.6)	0.130
Baseline LDH, U/L		253.5 (205.0–294.8)	251.0 (198.0–295.0)	249.0 (192.0–286.0)	266.0 (218.0–294.2)	0.437
Baseline ALP, U/L		173.0 (136.0–229.8)	171.0 (138.0–210.0)	163.0 (136.0–210.0)	202.5 (134.8–236.0)	0.414
Baseline albumin, g/L		41.8 (40.1–43.5)	42.9 (40.7–44.8)	41.4 (39.3–43.4)	40.8 (39.0–42.6)	<0.001
Baseline CRP, mg/L		5.7 (3.5–9.3)	5.7 (3.4–8.5)	5.3 (3.8–9.6)	5.9 (3.7–10.0)	0.610
Baseline ESR, mm/h		23.0 (15.2–31.0)	21.0 (15.0–30.0)	24.0 (14.0–32.0)	23.0 (17.8–32.0)	0.436
Neoadjuvant chemotherapy cycles		4.0 (3.0–4.0)	4.0 (3.0–4.0)	4.0 (3.0–4.0)	4.0 (3.0–4.0)	0.835
Sex	Female	75 (46.3%)	23 (35.4%)	23 (51.1%)	29 (55.8%)	0.067
	Male	87 (53.7%)	42 (64.6%)	22 (48.9%)	23 (44.2%)	
Primary site group	Axial/pelvic	17 (10.5%)	4 (6.2%)	7 (15.6%)	6 (11.5%)	0.274
	Extremity	145 (89.5%)	61 (93.8%)	38 (84.4%)	46 (88.5%)	
Baseline metastasis	0	147 (90.7%)	60 (92.3%)	41 (91.1%)	46 (88.5%)	0.772
	1	15 (9.3%)	5 (7.7%)	4 (8.9%)	6 (11.5%)	
Pathological fracture	0	142 (87.7%)	58 (89.2%)	41 (91.1%)	43 (82.7%)	0.401
	1	20 (12.3%)	7 (10.8%)	4 (8.9%)	9 (17.3%)	
Stage	IIA	59 (36.4%)	25 (38.5%)	19 (42.2%)	15 (28.8%)	0.682
	IIB	88 (54.3%)	35 (53.8%)	22 (48.9%)	31 (59.6%)	
	III	15 (9.3%)	5 (7.7%)	4 (8.9%)	6 (11.5%)	
NAC regimen	MAP	60 (37.0%)	25 (38.5%)	18 (40.0%)	17 (32.7%)	0.614
	AP	64 (39.5%)	28 (43.1%)	17 (37.8%)	19 (36.5%)	
	Other	38 (23.5%)	12 (18.5%)	10 (22.2%)	16 (30.8%)	
Dose delay during NAC	0	111 (68.5%)	49 (75.4%)	28 (62.2%)	34 (65.4%)	0.289
	1	51 (31.5%)	16 (24.6%)	17 (37.8%)	18 (34.6%)	
Dose reduction during NAC	0	138 (85.2%)	58 (89.2%)	39 (86.7%)	41 (78.8%)	0.276
	1	24 (14.8%)	7 (10.8%)	6 (13.3%)	11 (21.2%)	
G-CSF use during NAC	0	80 (49.4%)	36 (55.4%)	21 (46.7%)	23 (44.2%)	0.444
	1	82 (50.6%)	29 (44.6%)	24 (53.3%)	29 (55.8%)	
Antibiotic use during NAC	0	111 (68.5%)	45 (69.2%)	32 (71.1%)	34 (65.4%)	0.822
	1	51 (31.5%)	20 (30.8%)	13 (28.9%)	18 (34.6%)	
Systemic steroid use during NAC	0	85 (52.5%)	32 (49.2%)	27 (60.0%)	26 (50.0%)	0.491
	1	77 (47.5%)	33 (50.8%)	18 (40.0%)	26 (50.0%)	
Infection during NAC	0	131 (80.9%)	52 (80.0%)	40 (88.9%)	39 (75.0%)	0.217
	1	31 (19.1%)	13 (20.0%)	5 (11.1%)	13 (25.0%)	
Febrile neutropenia	0	140 (86.4%)	58 (89.2%)	37 (82.2%)	45 (86.5%)	0.573
	1	22 (13.6%)	7 (10.8%)	8 (17.8%)	7 (13.5%)	
Surgery type	amputation	28 (17.3%)	8 (12.3%)	8 (17.8%)	12 (23.1%)	0.308
	limb salvage	134 (82.7%)	57 (87.7%)	37 (82.2%)	40 (76.9%)	
Margin status	close	21 (13.0%)	10 (15.4%)	4 (8.9%)	7 (13.5%)	0.443
	negative	138 (85.2%)	55 (84.6%)	39 (86.7%)	44 (84.6%)	
	positive	3 (1.9%)	0 (0.0%)	2 (4.4%)	1 (1.9%)	

Data are median (IQR) or n (%). P values are descriptive and were calculated using Kruskal-Wallis tests for continuous variables and chi-square tests with Monte Carlo-estimated P values when expected cell counts were sparse for categorical variables. AP, doxorubicin plus cisplatin; MAP, high-dose methotrexate, doxorubicin, and cisplatin; G-CSF, granulocyte colony-stimulating factor; NAC, neoadjuvant chemotherapy.

Baseline clinical and treatment variables were generally similar across groups, although baseline albumin differed. T3 was obtained a median of 101 days after chemotherapy initiation and 3 days before surgery. The analysis therefore addresses preoperative treatment-response stratification among patients who reached surgery rather than early prediction during neoadjuvant treatment. Compared with the 26 otherwise eligible patients without complete T0-T3 data, included patients had smaller tumors on average; complete-case inclusion weighting did not materially change the primary association (**Supplementary Figure 1**; **Supplementary Table 14**).

### 3.2 Rule-derived peripheral inflammatory-lymphocyte recovery phenotype

**Figure 2:**
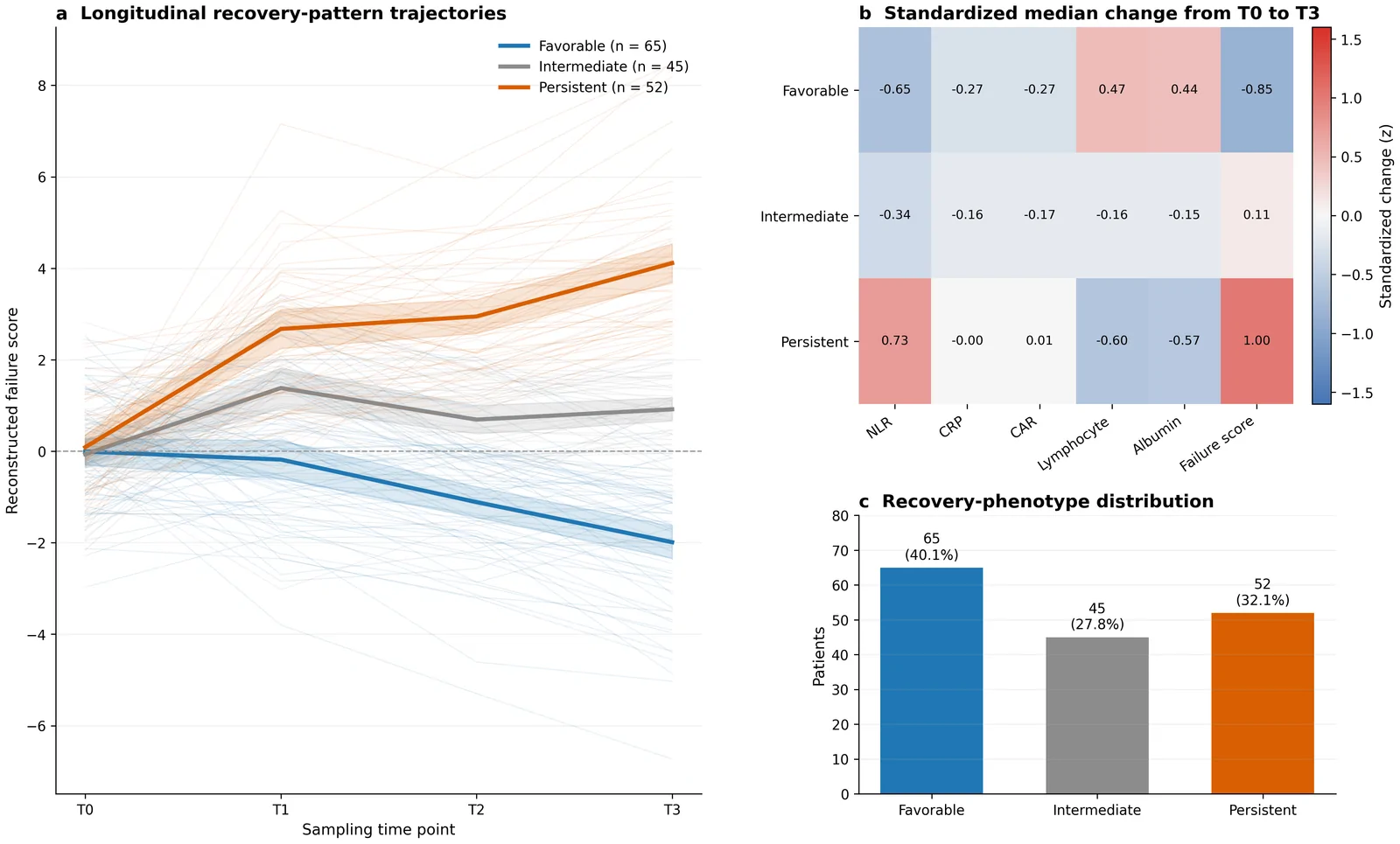
Treatment-period peripheral inflammatory-lymphocyte profiles. **(A)** Patient-level failure-score profiles from T0 to T3 by primary recovery phenotype; thin lines represent individual patients, thick lines group means, and shaded bands 95% confidence intervals. **(B)** Standardized median T0-to-T3 changes in NLR, CRP, CAR, lymphocyte count, albumin, and failure score by phenotype. **(C)** Distribution of the three phenotype groups. T1 and T2 are shown descriptively and were not used to fit a trajectory model or define the primary phenotype. Displayed component changes are expected partly by construction and do not constitute independent biological validation. NLR, neutrophil-to-lymphocyte ratio; CRP, C-reactive protein; CAR, C-reactive protein-to-albumin ratio.

Median failure scores at T0 were -0.11, -0.18, and 0.11 in the favorable, intermediate, and persistent groups, respectively. At T3, the corresponding values were -1.62, 0.98, and 3.87, with median T3-T0 changes of -1.76, 1.10, and 3.75 (**Figure 2**; **Table 1**). T1 and T2 profiles are shown descriptively; they did not determine the primary classification.

The persistent-failure group showed higher T3 NLR, SII, SIRI, and CAR and lower lymphocyte count, albumin, and PNI. These patterns were expected in part because the displayed variables contribute to the score and should be interpreted as a description of the rule rather than independent construct validation. Correlations and variance-inflation analyses confirmed substantial algebraic overlap, especially for PNI, while leave-one-component, leave-one-domain, and nonredundant primitive-variable analyses retained the direction of association (**Supplementary Tables S3; S4**; **Supplementary Figures 2, S3**).

Among 24 patients with paired immune-subset data, persistent failure was associated with lower baseline-adjusted T3 CD3+, CD4+, and CD8+ counts after Holm correction; NK-cell and B-cell comparisons were not significant after multiplicity adjustment. Because the subset was small and its pathological-response distribution differed meaningfully from patients without subset data, these findings are exploratory and susceptible to selection bias (**Supplementary Table 6**; **Supplementary Figure 4**). Phenotype distributions varied descriptively by chemotherapy regimen, but the global phenotype-by-regimen interaction was not detected (P for interaction=0.973), with wide regimen-specific uncertainty (**Supplementary Table 7**; **Supplementary Figure 5**).

### 3.3 Primary association with pathological response

**Figure 3:**
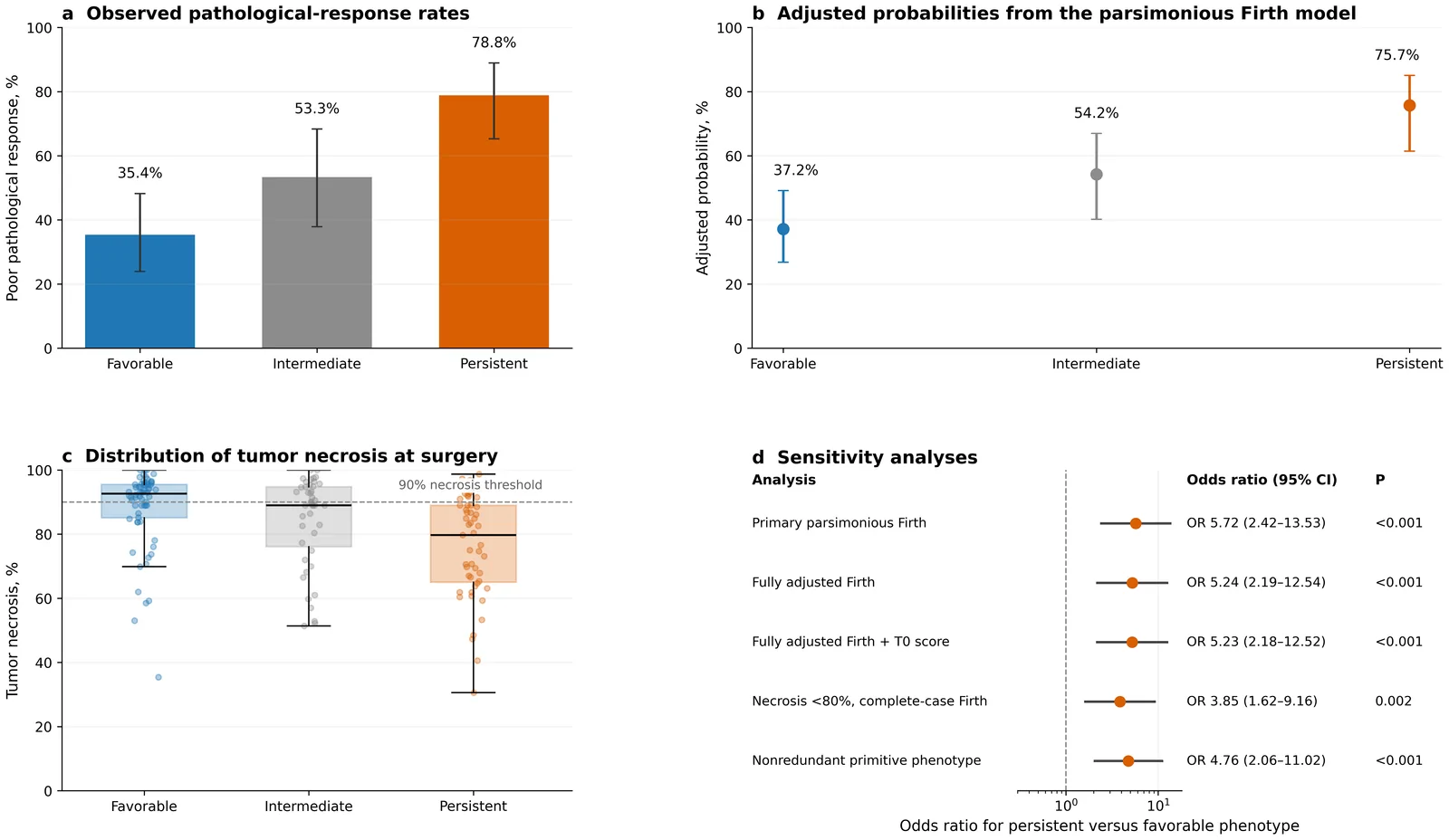
Recovery phenotype and pathological response. **(A)** Observed poor pathological response by phenotype. **(B)** Firth-model standardized probabilities with 95% confidence intervals. **(C)** Tumor necrosis percentage by phenotype; the dashed line marks 90% necrosis. **(D)** Table-style forest plot of the primary parsimonious Firth model and prespecified sensitivity analyses. Poor pathological response was defined as tumor necrosis <90%. The principal comparison was persistent recovery failure versus favorable recovery.

Poor pathological response occurred in 23 of 65 patients (35.4%) with favorable recovery, 24 of 45 (53.3%) with an intermediate phenotype, and 41 of 52 (78.8%) with persistent recovery failure. In the primary parsimonious Firth model, persistent failure was associated with poor pathological response versus favorable recovery (OR, 5.72; 95% CI, 2.42-13.53; P<0.001). The corresponding modified-Poisson adjusted RR was 2.10 (95% CI, 1.46-3.01; P<0.001) (**Figure 3**; **Table 4**).

**Table 4:** Primary association between recovery phenotype and poor pathological response, with adjusted relative and absolute effects.

Recovery phenotype	N	Poor response, n (%)	Firth OR (95% CI; P)	Adjusted RR (95% CI; P)	Standardized risk (95% CI)	Risk difference vs favorable (95% CI)
Favorable recovery	65	23 (35.4%)	Reference	Reference	37.2% (26.9-49.2)	Reference
Intermediate recovery phenotype	45	24 (53.3%)	2.07 (0.93-4.65); 0.076	1.51 (0.99-2.31); 0.054	54.2% (40.1-66.9)	17.0 pp (-1.7 to 33.4)
Persistent recovery failure	52	41 (78.8%)	5.72 (2.42-13.53); <0.001	2.10 (1.46-3.01); <0.001	75.7% (61.6-85.1)	38.6 pp (19.7 to 52.7)

Poor pathological response was tumor necrosis <90%. The primary parsimonious Firth model adjusted for age, sex, baseline metastasis, tumor size, and neoadjuvant regimen. Adjusted risk ratios were estimated using modified-Poisson regression with robust variance. Standardized risks and risk differences were derived from the primary Firth model. Favorable recovery was the reference.

Model-standardized risks were 37.2% (95% CI, 26.9%-49.2%) for favorable recovery, 54.2% (40.1%-66.9%) for the intermediate phenotype, and 75.7% (61.6%-85.1%) for persistent failure. The standardized risk difference for persistent failure versus favorable recovery was 38.6 percentage points (95% CI, 19.7-52.7). The intermediate-group Firth estimate did not meet the conventional significance threshold (OR, 2.07; 95% CI, 0.93-4.65; P = 0.076) (**Table 4**).

The fully adjusted Firth estimate remained similar (OR, 5.24; 95% CI, 2.19-12.54), as did a nonredundant primitive-variable phenotype (OR, 4.76; 95% CI, 2.06-11.02). Protocol-expanded and T3-only definitions produced ORs of 4.09 and 4.05, respectively. The 1,000-replicate full-pipeline bootstrap yielded a median OR of 5.70 (2.5th-97.5th percentile, 2.48-15.73), a median RR of 2.03 (1.46-3.02), and a median risk difference of 36.8 percentage points (20.2-53.8) (**Supplementary Tables 11 and S12**).

The association was also present across alternative pathological-response forms. For higher ordered Huvos response category, persistent failure had a common OR of 0.21 (95% CI, 0.10-0.43; P<0.001). Among 158 patients with an exact necrosis percentage, persistent failure was associated with 10.3 percentage points lower necrosis in robust linear regression (95% CI, -15.7 to -4.8; P<0.001) and 11.5 percentage points lower median necrosis (95% CI, -17.1 to -6.0; P<0.001). The complete-case <80% analysis was directionally consistent (**Supplementary Table 10**).

### 3.4 Exploratory event-related outcomes

**Figure 4:**
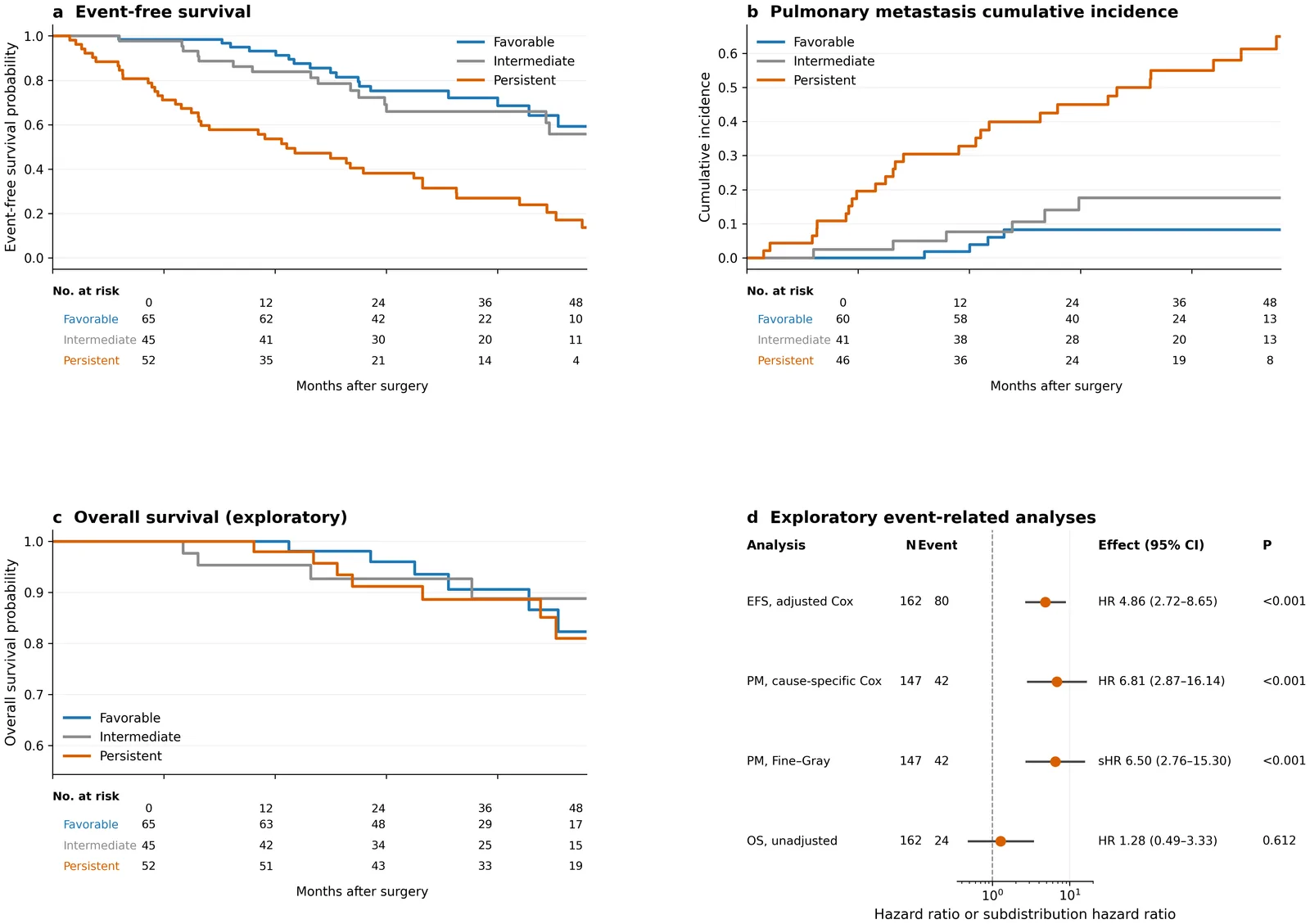
Exploratory event-related analyses. **(A)** Kaplan-Meier curves for EFS. **(B)** Aalen-Johansen cumulative incidence of pulmonary metastasis, treating death before pulmonary metastasis as a competing event. **(C)** Kaplan-Meier curves for OS. Numbers at risk are shown below panels **(A–C)**. **(D)** Table-style forest plot of parsimonious EFS and pulmonary-metastasis cause-specific Cox models, the pulmonary-metastasis Fine-Gray model, and unadjusted OS. All analyses in this figure are exploratory; pulmonary-metastasis analyses were restricted to patients without metastasis at diagnosis, and OS was immature with 24 deaths.

In exploratory parsimonious models, persistent failure was associated with EFS (HR, 4.86; 95% CI, 2.72-8.65) and the cause-specific hazard of pulmonary metastasis (HR, 6.81; 95% CI, 2.87-16.14). The pulmonary-metastasis Fine-Gray model was directionally consistent (sHR, 6.50; 95% CI, 2.76-15.30) (**Figure 4**; **Table 2**; **Supplementary Table 9**). These estimates were secondary, based on relatively few events, and were not interpreted as validated prognostic effects.

Parsimonious proportional-hazards diagnostics did not show a significant global violation for EFS (P = 0.366) or pulmonary metastasis (P = 0.491), although the pulmonary-metastasis persistent-failure term approached the threshold (P = 0.052). Fixed-horizon and time-specific analyses showed larger early estimates and attenuation with longer follow-up. OS was not associated with phenotype in the unadjusted model (persistent failure HR, 1.28; 95% CI, 0.49-3.33; P = 0.612) and remained immature with 24 deaths (**Supplementary Table 8**).

### 3.5 Internal validation and model comparison

**Figure 5:**
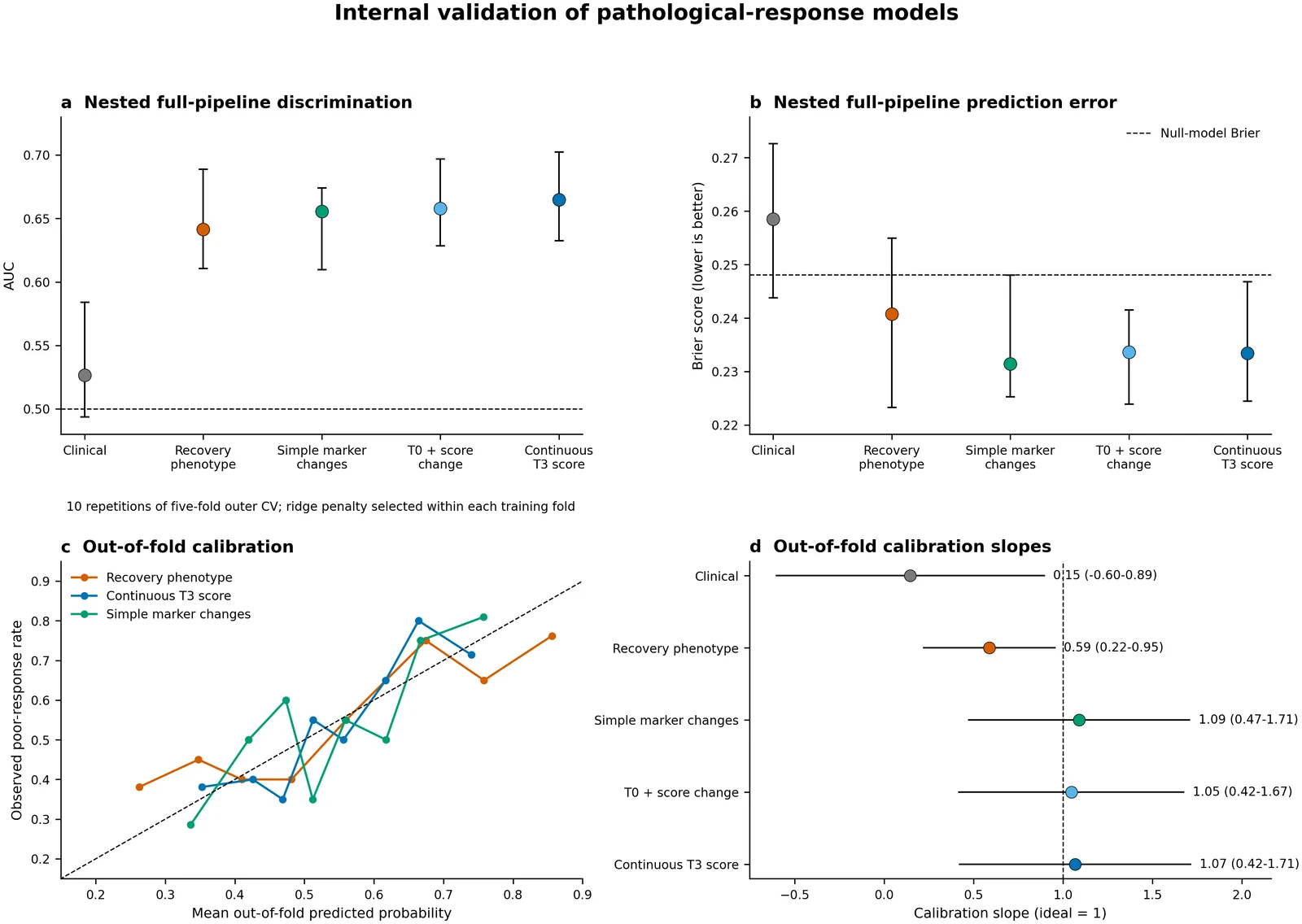
Nested full-pipeline internal validation and out-of-fold calibration. **(A)** Mean AUC across 10 repeats of five-fold outer cross-validation. **(B)** Mean Brier score. **(C)** Mean out-of-fold calibration curves. **(D)** Calibration slopes with 95% confidence intervals. In each outer training fold, T0 standardization, scores, distributional thresholds, phenotype assignment, preprocessing, and ridge-penalty selection were re-estimated before held-out prediction. The categorical recovery phenotype did not outperform the continuous T3 score, and its out-of-fold calibration slope indicated residual overfitting. These analyses are internal validation and do not constitute external validation.

In nested full-pipeline repeated cross-validation, mean AUCs were 0.527 for the clinical model, 0.641 for the recovery-phenotype model, 0.656 for simple marker changes, 0.658 for T0 plus change, and 0.665 for the continuous T3 score. Corresponding Brier scores were 0.259, 0.241, 0.231, 0.234, and 0.233 (**Figure 5**; **Supplementary Table 5**). The phenotype therefore did not outperform the continuous T3 score or simpler change-based models. Its mean out-of-fold calibration slope was 0.587 (95% CI, 0.220-0.953); across the 10 repeats, slopes ranged from 0.296 to 0.739, indicating residual overfitting (**Supplementary Table 5**).

Adding a T1/T2-derived longitudinal slope to the continuous T3 model did not provide statistically detectable incremental information (likelihood-ratio P = 0.158). In internal time-stratified analyses, the phenotype used alone retained similar group assignments and moderate discrimination in later calendar periods, and the phenotype-by-era interaction was not significant (P = 0.705). However, multivariable prediction probabilities combining phenotype and clinical variables transported inconsistently across calendar splits. These analyses were internal calendar-time analyses and do not constitute external validation (**Supplementary Tables 11 and S13**).

## 4 Discussion

The principal finding is a robust association between a rule-derived preoperative peripheral inflammatory-lymphocyte recovery phenotype and pathological response among patients with osteosarcoma who completed neoadjuvant chemotherapy and underwent definitive surgery. Persistent recovery failure was associated with approximately twice the adjusted risk of poor pathological response and a 38.6-percentage-point higher standardized risk than favorable recovery. The association persisted across full-pipeline bootstrap, alternative classification rules, nonredundant scoring, ordered Huvos grade, and continuous necrosis analyses. By contrast, the categorical phenotype did not show predictive superiority over the continuous T3 score, and its out-of-fold calibration indicated residual overfitting.

The timing of measurement determines the appropriate clinical interpretation. T3 was obtained a median of 3 days before surgery, when pathological response had already developed biologically but remained unavailable clinically. The phenotype should therefore be considered a preoperative treatment-response stratifier, not an early-treatment predictor. This interpretation complements the established role of histological response after resection (4, 5) but does not justify delaying surgery or changing treatment on the basis of the current results.

Previous osteosarcoma studies have mainly evaluated single inflammatory or nutritional indices. Pretreatment NLR and PLR have been associated with prognosis (14), and related leukocyte ratios have shown diagnostic or prognostic associations in other cohorts (15). PNI and SII have also been examined as survival biomarkers (16), while a recent meta-analysis emphasized inconsistency across studies (18). The present work does not establish a superior composite biomarker; it examines whether a transparent combined preoperative pattern is associated with pathological response.

Treatment-period change remains relevant because dynamic NLR has previously added prognostic information beyond baseline values in osteosarcoma (19). In the present study, however, nested full-pipeline cross-validation showed that the categorical phenotype did not outperform the continuous T3 score, T0 plus change, or a simpler marker-change model. Its practical contribution is therefore interpretability as a three-level risk stratifier rather than demonstrated incremental predictive accuracy.

The peripheral pattern may reflect several overlapping processes rather than a single immunological mechanism. The broader immune-resilience framework concerns favorable immune function under inflammatory stress (20), while chemotherapy can alter tumor-cell death, antigen presentation, and immune-cell dynamics (23). Cancer-associated lymphopenia may also accompany impaired antitumor responses or treatment intolerance (24). Nevertheless, routine blood counts, CRP, albumin, and PNI cannot distinguish immune competence from chemotherapy toxicity, infection, nutritional change, tumor burden, or supportive care. The present findings therefore support an inflammatory-lymphocyte phenotype, not functional immune-resilience validation.

The direction of the association is compatible with a myeloid-skewed tumor-host context described in osteosarcoma and other cancers (9, 10, 25). Neutrophils and related myeloid programs can participate in invasion, immune escape, and metastatic progression (26, 27), and neutrophil extracellular traps have been associated with poor neoadjuvant response in pediatric osteosarcoma (28). However, the composite repeatedly represents several primitive variables: lymphocyte count contributes to NLR, SII, SIRI, and PNI, while albumin contributes to CAR and PNI. The high collinearity and expected component differences mean that **Figure 2** and **Table 1** describe the classification rule rather than independently validating its biology.

The paired immune-subset findings provide limited supportive evidence. After baseline adjustment and Holm correction, persistent failure was associated with lower T3 CD3+, CD4+, and CD8+ counts, whereas NK-cell and B-cell results were not significant. This direction is compatible with the heterogeneous immune states described in osteosarcoma (7, 8, 29), but only 24 patients had paired subset measurements and availability was not random. Peripheral counts also cannot represent the intratumoral immune compartment. These findings should remain exploratory and should not be described as mechanistic validation.

The event-related analyses are hypothesis-generating. Persistent failure was associated with EFS and pulmonary metastasis in cause-specific and competing-risk analyses, but event numbers were limited and estimates were imprecise. The effect was larger at early fixed horizons and attenuated over longer follow-up, so a single HR should not be interpreted as a constant long-term effect. Further adjustment for pathological response attenuated the associations, suggesting shared information with treatment sensitivity. OS was immature with 24 deaths and did not support a multivariable claim.

The phenotype also should not be conflated with checkpoint sensitivity. Pembrolizumab showed limited activity in unselected bone sarcoma in SARC028 (11), and nivolumab-based treatment has not established consistent activity in osteosarcoma (12). Correlative SARC028 analyses linked response to features of the tumor immune infiltrate (13), which cannot be inferred from this peripheral composite. The current phenotype may guide hypotheses for future paired peripheral and tumor studies, but it cannot select immunotherapy or substitute for tissue-level profiling.

A further distinction is required between stability of phenotypic stratification and transportability of a prediction model. When early-period scoring parameters were applied to later patients, phenotype-only discrimination and group agreement were reasonably stable, with no detectable phenotype-by-era interaction. In contrast, multivariable models combining the phenotype with clinical variables had inconsistent time-split performance. These calendar splits were internal calendar-time analyses and do not constitute external validation. Future work should use prospectively fixed laboratory windows and rules, independent multicenter cohorts, and biological systems capable of linking peripheral patterns to tumor response. Patient-derived three-dimensional tumor cultures may support functional drug-response testing, while broader neuro-immune-tumor communication frameworks illustrate the need to study host biology beyond isolated blood ratios (30, 31).

Study strengths include a clinically established pathological endpoint, explicit score specification, a transparent primary patient-level rule, Firth estimation for the primary association, reporting of relative and absolute effects, full-pipeline bootstrap, nested cross-validation without information leakage, direct benchmarking against simpler biomarkers, and robustness across ordered and continuous pathological-response forms. The analysis also explicitly quantified algebraic overlap, immune-subset selection, regimen interaction, competing risks, and proportional-hazards diagnostics.

Several limitations constrain interpretation. This was a single-center retrospective analysis restricted to patients who completed neoadjuvant chemotherapy and reached surgery with valid T0 and T3 data; it cannot be generalized to patients who progressed, discontinued treatment, or did not undergo surgery. The 0.05 reserve-change boundary is an operational convention within an identifiable interval rather than a unique prespecified or biological threshold. Residual and unmeasured confounding remain possible despite extensive sensitivity analyses. The phenotype is dominated by preoperative T3 status, and T1/T2 did not show clear incremental value. Component reuse creates substantial collinearity. Internal validation showed residual overfitting, while multivariable calendar-split prediction was unstable. Immune-subset analyses were small and selected. Finally, pulmonary-metastasis events were limited and OS was immature. Predictive superiority, external validity, biological mechanism, and clinical utility were not established, and all event-related analyses remain exploratory.

In summary, the rule-derived preoperative peripheral inflammatory-lymphocyte recovery phenotype was robustly associated with pathological response in resected osteosarcoma. Its three-level form may provide an interpretable description of preoperative host inflammatory and lymphocyte-nutritional status, but it did not outperform the continuous T3 score and should not be treated as a validated prediction model, functional immune construct, or clinical decision tool. Independent prospective validation with fixed rules and integrated peripheral and tumor measurements is required.

## 5 Conclusion

A rule-derived T0-T3 preoperative peripheral inflammatory-lymphocyte recovery phenotype was associated with poor pathological response among patients with osteosarcoma who completed neoadjuvant chemotherapy and underwent definitive surgery. The association was robust across alternative classifications and pathological-response definitions.

## Data Availability

The original contributions presented in the study are included in the article/**Supplementary Material**. Further inquiries can be directed to the corresponding author/s.
