# Supplementary material for: Rule-derived preoperative inflammatory-lymphocyte recovery phenotype and pathological response in resected osteosarcoma: a retrospective cohort study
Source: Front Immunol. 2026 Sep 7;17:1925540. doi: 10.3389/fimmu.2026.1925540 (PMC13590790; doi:10.3389/fimmu.2026.1925540)
Supplement: Supplementary file 1 [file Table1.docx]

Supplementary Materials

# Supplementary Tables

## Supplementary Table S1. Score construction and T0 standardization parameters

**Panel A. Score formula and component weights.**

| **component** | **source column** | **transformation** | **domain** | **domain weight** | **failure score weight** |
| --- | --- | --- | --- | --- | --- |
| lnNLR | NLR | Natural log | Myeloid-inflammatory burden | 0.250 | 0.250 |
| lnSII | SII | Natural log | Myeloid-inflammatory burden | 0.250 | 0.250 |
| lnSIRI | SIRI | Natural log | Myeloid-inflammatory burden | 0.250 | 0.250 |
| lnCAR | CAR | Natural log | Myeloid-inflammatory burden | 0.250 | 0.250 |
| Lymphocyte | lymphocyte_10e9_L | Untransformed | Lymphocyte-nutritional reserve | 0.333 | -0.333 |
| Albumin | albumin_g_L | Untransformed | Lymphocyte-nutritional reserve | 0.333 | -0.333 |
| PNI | PNI | Untransformed | Lymphocyte-nutritional reserve | 0.333 | -0.333 |

**Panel B. T0 reference parameters for score standardization.**

| **component** | **source column** | **transformation** | **domain** | **domain weight** | **failure score weight** | **T0 reference mean** | **T0 reference SD** | **reference N** |
| --- | --- | --- | --- | --- | --- | --- | --- | --- |
| lnNLR | NLR | Natural log | Myeloid-inflammatory burden | 0.250 | 0.250 | 0.837 | 0.241 | 181 |
| lnSII | SII | Natural log | Myeloid-inflammatory burden | 0.250 | 0.250 | 6.456 | 0.317 | 181 |
| lnSIRI | SIRI | Natural log | Myeloid-inflammatory burden | 0.250 | 0.250 | -0.022 | 0.351 | 181 |
| lnCAR | CAR | Natural log | Myeloid-inflammatory burden | 0.250 | 0.250 | -2.028 | 0.594 | 181 |
| Lymphocyte | lymphocyte_10e9_L | Untransformed | Lymphocyte-nutritional reserve | 0.333 | -0.333 | 1.624 | 0.319 | 181 |
| Albumin | albumin_g_L | Untransformed | Lymphocyte-nutritional reserve | 0.333 | -0.333 | 42.143 | 2.044 | 181 |
| PNI | PNI | Untransformed | Lymphocyte-nutritional reserve | 0.333 | -0.333 | 50.257 | 2.593 | 181 |

## Supplementary Table S2. Primary recovery-phenotype rule and numeric thresholds

**Panel A. Primary recovery-phenotype rule.**

| **classification** | **primary rule** | **threshold origin** | **prespecification status** | **analysis role** |
| --- | --- | --- | --- | --- |
| Favorable recovery | T3 failure score below the cohort median AND [T3-T0 failure-score change <= -0.25 OR (T0 failure score below the lower tertile AND T3 failure score below the lower tertile)] | The -0.25 failure-score decrease was part of the original operational definition; all distributional cut points are estimated from the analysis cohort without using the outcome. | Original operational rule | Primary analysis definition |
| Persistent recovery failure | T3 failure score at or above the upper-tertile cut point AND T3-T0 reserve-score change < 0.05 | The 0.05 reserve-change boundary was defined as a transparent operational convention within the interval compatible with the rounded patient-level phenotype-assignment indicator; it was not documented in the study protocol and was not selected using pathological-response or survival outcomes. | Post hoc implementation threshold, transparently disclosed | Primary analysis definition; alternative definitions are sensitivity analyses |
| Intermediate recovery phenotype | Does not meet the primary favorable-recovery or persistent-failure rules | Residual category after applying the two mutually exclusive rules | Original operational rule | Primary analysis definition |

**Panel B. Numeric thresholds, identifiable interval, and threshold origins.**

| **parameter** | **value** | **operator** | **threshold origin** | **prespecification status** | **analysis role** |
| --- | --- | --- | --- | --- | --- |
| T3 median failure score | 0.702 | < | Analysis-cohort distribution, estimated without outcome information | Original operational or distribution-based rule | Primary analysis classification |
| T3 upper-tertile cut point | 2.067 | >= | Analysis-cohort distribution, estimated without outcome information | Original operational or distribution-based rule | Primary analysis classification |
| T0 lower-tertile cut point | -0.341 | < | Analysis-cohort distribution, estimated without outcome information | Original operational or distribution-based rule | Primary analysis classification |
| T3 lower-tertile cut point | -0.844 | < | Analysis-cohort distribution, estimated without outcome information | Original operational or distribution-based rule | Primary analysis classification |
| Required failure-score decrease | -0.250 | <= | Original operational rule | Original operational or distribution-based rule | Primary analysis classification |
| No meaningful reserve-recovery cut point | 0.050 | < | Transparent interior operational convention within the interval compatible with the rounded patient-level assignment indicator (>0.037 to ≤0.057); not outcome-optimized | Post hoc operational implementation threshold; not uniquely identifiable from rounded data | Primary analysis classification |

Note: The 0.05 boundary is an operational convention within the identifiable interval; it is not a prespecified biological threshold.

## Supplementary Table S3. Component correlations and collinearity diagnostics

| **time point** | **component** | **N** | **VIF** |
| --- | --- | --- | --- |
| T0 | lnNLR | 162 | 3.36 |
| T0 | lnSII | 162 | 2.17 |
| T0 | lnSIRI | 162 | 2.05 |
| T0 | lnCAR | 162 | 1.04 |
| T0 | Lymphocyte | 162 | 1299.45 |
| T0 | Albumin | 162 | 2124.58 |
| T0 | PNI | 162 | 3612.58 |
| T3 | lnNLR | 162 | 8.10 |
| T3 | lnSII | 162 | 4.86 |
| T3 | lnSIRI | 162 | 5.10 |
| T3 | lnCAR | 162 | 1.27 |
| T3 | Lymphocyte | 162 | 3969.12 |
| T3 | Albumin | 162 | 7541.54 |
| T3 | PNI | 162 | 14021.73 |

## Supplementary Table S4. Leave-one-component, leave-one-domain, and nonredundant primitive-variable analyses

| **model** | **term** | **estimate log odds** | **OR** | **95% CI low** | **95% CI high** | **p value** | **N** | **events** | **outcome** | **favorable N** | **intermediate N** | **persistent N** |
| --- | --- | --- | --- | --- | --- | --- | --- | --- | --- | --- | --- | --- |
| Leave out lnNLR | Persistent recovery failure vs favorable recovery | 1.923 | 6.841 | 2.780 | 16.833 | <0.001 | 162 | 88 | Poor pathological response | 65 | 45 | 52 |
| Leave out lnSII | Persistent recovery failure vs favorable recovery | 1.754 | 5.775 | 2.367 | 14.092 | <0.001 | 162 | 88 | Poor pathological response | 63 | 47 | 52 |
| Leave out lnSIRI | Persistent recovery failure vs favorable recovery | 1.840 | 6.297 | 2.590 | 15.311 | <0.001 | 162 | 88 | Poor pathological response | 67 | 43 | 52 |
| Leave out lnCAR | Persistent recovery failure vs favorable recovery | 1.663 | 5.274 | 2.155 | 12.904 | <0.001 | 162 | 88 | Poor pathological response | 68 | 44 | 50 |
| Leave out Lymphocyte | Persistent recovery failure vs favorable recovery | 2.000 | 7.391 | 2.911 | 18.769 | <0.001 | 162 | 88 | Poor pathological response | 64 | 47 | 51 |
| Leave out Albumin | Persistent recovery failure vs favorable recovery | 1.557 | 4.747 | 2.029 | 11.107 | <0.001 | 162 | 88 | Poor pathological response | 69 | 40 | 53 |
| Leave out PNI | Persistent recovery failure vs favorable recovery | 1.798 | 6.036 | 2.482 | 14.681 | <0.001 | 162 | 88 | Poor pathological response | 68 | 42 | 52 |
| Myeloid-inflammatory domain only | Persistent recovery failure vs favorable recovery | 1.395 | 4.036 | 1.693 | 9.623 | 0.002 | 162 | 88 | Poor pathological response | 67 | 42 | 53 |
| Lymphocyte-nutritional reserve domain only | Persistent recovery failure vs favorable recovery | 1.408 | 4.089 | 1.725 | 9.691 | 0.001 | 162 | 88 | Poor pathological response | 59 | 49 | 54 |

## Supplementary Table S5. Nested cross-validation, out-of-fold calibration, and model comparisons

**Panel A. Nested full-pipeline cross-validation performance.**

| **model** | **repeats** | **AUC mean** | **AUC SD** | **AUC q025** | **AUC q975** | **Brier mean** | **Brier SD** | **Brier q025** | **Brier q975** |
| --- | --- | --- | --- | --- | --- | --- | --- | --- | --- |
| Clinical | 10 | 0.527 | 0.033 | 0.494 | 0.584 | 0.259 | 0.010 | 0.244 | 0.273 |
| Continuous T3 score | 10 | 0.665 | 0.024 | 0.632 | 0.702 | 0.233 | 0.007 | 0.224 | 0.247 |
| Recovery phenotype | 10 | 0.641 | 0.026 | 0.611 | 0.689 | 0.241 | 0.010 | 0.223 | 0.255 |
| Simple marker changes | 10 | 0.656 | 0.022 | 0.610 | 0.674 | 0.231 | 0.008 | 0.225 | 0.248 |
| T0 plus change | 10 | 0.658 | 0.025 | 0.628 | 0.697 | 0.234 | 0.006 | 0.224 | 0.242 |

**Panel B. Out-of-fold performance, calibration, and repeat-specific calibration stability.**

| **model** | **OOF AUC** | **OOF Brier** | **scaled Brier** | **null Brier** | **calibration intercept** | **intercept 95% CI low** | **intercept 95% CI high** | **calibration slope** | **slope 95% CI low** | **slope 95% CI high** | **slope mean** | **slope median** | **slope min** | **slope max** |
| --- | --- | --- | --- | --- | --- | --- | --- | --- | --- | --- | --- | --- | --- | --- |
| Clinical | 0.526 | 0.255 | -0.027 | 0.248 | 0.147 | -0.190 | 0.484 | 0.145 | -0.603 | 0.894 | 0.151 | 0.056 | -0.231 | 0.724 |
| Continuous T3 score | 0.667 | 0.230 | 0.071 | 0.248 | -0.006 | -0.341 | 0.329 | 1.067 | 0.422 | 1.713 | 0.990 | 0.968 | 0.341 | 1.653 |
| Recovery phenotype | 0.646 | 0.236 | 0.049 | 0.248 | 0.055 | -0.270 | 0.381 | 0.587 | 0.220 | 0.953 | 0.512 | 0.495 | 0.296 | 0.739 |
| Simple marker changes | 0.657 | 0.228 | 0.079 | 0.248 | -0.011 | -0.347 | 0.324 | 1.089 | 0.472 | 1.707 | 1.023 | 1.087 | 0.396 | 1.405 |
| T0 plus change | 0.664 | 0.231 | 0.071 | 0.248 | -0.001 | -0.335 | 0.334 | 1.045 | 0.417 | 1.674 | 0.892 | 0.869 | 0.548 | 1.317 |

**Panel C. Recovery phenotype versus continuous T3 score.**

| **comparison** | **delta AUC** | **delta AUC 95% CI low** | **delta AUC 95% CI high** | **delta Brier T3 minus phenotype** | **delta Brier 95% CI low** | **delta Brier 95% CI high** |
| --- | --- | --- | --- | --- | --- | --- |
| Recovery phenotype minus continuous T3 score | -0.021 | -0.054 | 0.014 | -0.006 | -0.020 | 0.008 |

Note: The mean OOF slope uses each patient's mean OOF prediction across repeats. Repeat-specific slope ranges quantify variation across independent resampling splits.

## Supplementary Table S6. Exploratory immune-subset findings and selection audit

**Panel A. Baseline-adjusted immune-subset analysis with multiplicity correction.**

| **marker** | **paired N** | **contrast** | **adjusted geometric mean ratio** | **95% CI low** | **95% CI high** | **p value** | **contrast display** | **Holm adjusted p within contrast** | **Holm adjusted p all tests** | **FDR adjusted p all tests** |
| --- | --- | --- | --- | --- | --- | --- | --- | --- | --- | --- |
| CD3+ T cells | 24 | Intermediate recovery phenotype | 0.677 | 0.498 | 0.921 | 0.016 | Intermediate recovery phenotype | 0.063 | 0.094 | 0.031 |
| CD3+ T cells | 24 | Persistent recovery failure | 0.579 | 0.440 | 0.763 | <0.001 | Persistent recovery failure | 0.003 | 0.005 | 0.005 |
| CD4+ T cells | 24 | Intermediate recovery phenotype | 0.616 | 0.432 | 0.878 | 0.010 | Intermediate recovery phenotype | 0.049 | 0.069 | 0.025 |
| CD4+ T cells | 24 | Persistent recovery failure | 0.603 | 0.439 | 0.829 | 0.003 | Persistent recovery failure | 0.014 | 0.031 | 0.017 |
| CD8+ T cells | 24 | Intermediate recovery phenotype | 0.690 | 0.462 | 1.031 | 0.068 | Intermediate recovery phenotype | 0.205 | 0.273 | 0.097 |
| CD8+ T cells | 24 | Persistent recovery failure | 0.586 | 0.411 | 0.836 | 0.005 | Persistent recovery failure | 0.016 | 0.042 | 0.017 |
| NK cells | 24 | Intermediate recovery phenotype | 0.762 | 0.433 | 1.343 | 0.329 | Intermediate recovery phenotype | 0.658 | 0.666 | 0.345 |
| NK cells | 24 | Persistent recovery failure | 0.607 | 0.370 | 0.994 | 0.048 | Persistent recovery failure | 0.095 | 0.239 | 0.080 |
| B cells | 24 | Intermediate recovery phenotype | 0.701 | 0.326 | 1.508 | 0.345 | Intermediate recovery phenotype | 0.658 | 0.666 | 0.345 |
| B cells | 24 | Persistent recovery failure | 0.662 | 0.335 | 1.310 | 0.222 | Persistent recovery failure | 0.222 | 0.666 | 0.278 |

**Panel B. Immune-subset availability and selection audit.**

| **variable** | **level** | **with subset N** | **without subset N** | **with subset mean** | **without subset mean** | **standardized mean difference** | **p value** | **with subset proportion** | **without subset proportion** | **absolute SMD** | **material imbalance SMD ge 0 20** |
| --- | --- | --- | --- | --- | --- | --- | --- | --- | --- | --- | --- |
| age |  | 24 | 138 | 22.958 | 21.507 | 0.154 | 0.495 |  |  | 0.154 | No |
| Tumor size (cm) |  | 24 | 138 | 9.258 | 8.598 | 0.228 | 0.318 |  |  | 0.228 | Yes |
| Baseline LDH (U/L) |  | 24 | 138 | 258.333 | 250.913 | 0.111 | 0.652 |  |  | 0.111 | No |
| Baseline ALP (U/L) |  | 24 | 138 | 185.667 | 193.543 | -0.094 | 0.670 |  |  | 0.094 | No |
| Neoadjuvant chemotherapy cycles |  | 24 | 138 | 3.583 | 3.601 | -0.022 | 0.929 |  |  | 0.022 | No |
| Baseline failure score |  | 24 | 138 | -0.256 | 0.054 | -0.288 | 0.226 |  |  | 0.288 | Yes |
| sex | Female | 24 | 138 |  |  | -0.412 | 0.079 | 0.292 | 0.493 | 0.412 | Yes |
| sex | Male | 24 | 138 |  |  | 0.412 | 0.079 | 0.708 | 0.507 | 0.412 | Yes |
| Baseline metastasis | 0 | 24 | 138 |  |  | 0.038 | 1.000 | 0.917 | 0.906 | 0.038 | No |
| Baseline metastasis | 1 | 24 | 138 |  |  | -0.038 | 1.000 | 0.083 | 0.094 | 0.038 | No |
| Regimen group | AP | 24 | 138 |  |  | -0.048 | 1.000 | 0.375 | 0.399 | 0.048 | No |
| Regimen group | MAP | 24 | 138 |  |  | -0.303 | 0.253 | 0.250 | 0.391 | 0.303 | Yes |
| Regimen group | Other | 24 | 138 |  |  | 0.362 | 0.114 | 0.375 | 0.210 | 0.362 | Yes |
| Recovery phenotype | Favorable recovery | 24 | 138 |  |  | -0.063 | 0.825 | 0.375 | 0.406 | 0.063 | No |
| Recovery phenotype | Intermediate recovery phenotype | 24 | 138 |  |  | -0.074 | 0.811 | 0.250 | 0.283 | 0.074 | No |
| Recovery phenotype | Persistent recovery failure | 24 | 138 |  |  | 0.134 | 0.636 | 0.375 | 0.312 | 0.134 | No |
| Poor pathological response | 0 | 24 | 138 |  |  | 0.395 | 0.080 | 0.625 | 0.428 | 0.395 | Yes |
| Poor pathological response | 1 | 24 | 138 |  |  | -0.395 | 0.080 | 0.375 | 0.572 | 0.395 | Yes |
| EFS event | 0 | 24 | 138 |  |  | 0.083 | 0.826 | 0.542 | 0.500 | 0.083 | No |
| EFS event | 1 | 24 | 138 |  |  | -0.083 | 0.826 | 0.458 | 0.500 | 0.083 | No |

Note: The immune-subset analyses are exploratory because only 24 patients had paired measurements and availability was not random.

## Supplementary Table S7. Chemotherapy-regimen distributions, pathological-response rates, and phenotype-by-regimen interaction

**Panel A. Poor pathological-response rates by chemotherapy regimen and recovery phenotype.**

| **Regimen** | **Recovery phenotype** | **N** | **Poor-response events** | **Poor-response rate** |
| --- | --- | --- | --- | --- |
| MAP | Favorable recovery | 25 | 11 | 44.0% |
| MAP | Intermediate recovery phenotype | 18 | 11 | 61.1% |
| MAP | Persistent recovery failure | 17 | 14 | 82.4% |
| AP | Favorable recovery | 28 | 9 | 32.1% |
| AP | Intermediate recovery phenotype | 17 | 9 | 52.9% |
| AP | Persistent recovery failure | 19 | 14 | 73.7% |
| Other | Favorable recovery | 12 | 3 | 25.0% |
| Other | Intermediate recovery phenotype | 10 | 4 | 40.0% |
| Other | Persistent recovery failure | 16 | 13 | 81.3% |

**Panel B. Recovery-phenotype distribution within each chemotherapy regimen.**

| **Regimen** | **Total N** | **Favorable recovery** | **Intermediate phenotype** | **Persistent recovery failure** |
| --- | --- | --- | --- | --- |
| MAP | 60 | 25 (41.7%) | 18 (30.0%) | 17 (28.3%) |
| AP | 64 | 28 (43.8%) | 17 (26.6%) | 19 (29.7%) |
| Other | 38 | 12 (31.6%) | 10 (26.3%) | 16 (42.1%) |

**Panel C. Global distribution and phenotype-by-regimen interaction tests.**

| **Test** | **Chi-square** | **df** | **P value** | **Interpretation** |
| --- | --- | --- | --- | --- |
| Phenotype distribution across regimens | 2.673 | 4 | 0.614 | Descriptive chi-square test |
| Phenotype-by-regimen interaction | 0.504 | 4 | 0.973 | Likelihood-ratio test; P for interaction |

Note: MAP denotes high-dose methotrexate, doxorubicin, and cisplatin; AP denotes doxorubicin plus cisplatin. Poor-response values are n/N (%). The interaction model contained recovery phenotype, regimen, and phenotype-by-regimen interaction terms with the same prespecified adjustment set used for the primary analysis. P for interaction is displayed once for the global regimen category.

## Supplementary Table S8. Exploratory time-dependent analyses and proportional-hazards diagnostics

**Panel A. Time-dependent exploratory EFS analyses.**

| **contrast** | **time months** | **HR** | **95% CI low** | **95% CI high** |
| --- | --- | --- | --- | --- |
| Intermediate recovery phenotype vs favorable recovery | 6 | 1.519 | 0.343 | 6.739 |
| Intermediate recovery phenotype vs favorable recovery | 12 | 1.274 | 0.482 | 3.370 |
| Intermediate recovery phenotype vs favorable recovery | 24 | 1.058 | 0.547 | 2.045 |
| Intermediate recovery phenotype vs favorable recovery | 36 | 0.946 | 0.454 | 1.970 |
| Persistent recovery failure vs favorable recovery | 6 | 11.728 | 3.338 | 41.199 |
| Persistent recovery failure vs favorable recovery | 12 | 7.557 | 3.358 | 17.006 |
| Persistent recovery failure vs favorable recovery | 24 | 4.750 | 2.562 | 8.804 |
| Persistent recovery failure vs favorable recovery | 36 | 3.596 | 1.704 | 7.586 |

**Panel B. Parsimonious proportional-hazards diagnostics.**

| **model** | **term** | **correlation with log event time** | **p value** | **N** | **events** | **diagnostic** |
| --- | --- | --- | --- | --- | --- | --- |
| Exploratory EFS parsimonious cause-specific Cox | Intermediate recovery phenotype vs favorable recovery | 0.049 | 0.666 | 162 | 80 | Schoenfeld-residual correlation with log event time |
| Exploratory EFS parsimonious cause-specific Cox | Persistent recovery failure vs favorable recovery | -0.188 | 0.095 | 162 | 80 | Schoenfeld-residual correlation with log event time |
| Exploratory EFS parsimonious cause-specific Cox | age | -0.159 | 0.159 | 162 | 80 | Schoenfeld-residual correlation with log event time |
| Exploratory EFS parsimonious cause-specific Cox | Male vs female | -0.106 | 0.351 | 162 | 80 | Schoenfeld-residual correlation with log event time |
| Exploratory EFS parsimonious cause-specific Cox | Baseline metastasis | 0.075 | 0.511 | 162 | 80 | Schoenfeld-residual correlation with log event time |
| Exploratory EFS parsimonious cause-specific Cox | Tumor size (cm) | 0.102 | 0.370 | 162 | 80 | Schoenfeld-residual correlation with log event time |
| Exploratory EFS parsimonious cause-specific Cox | AP vs MAP | -0.108 | 0.342 | 162 | 80 | Schoenfeld-residual correlation with log event time |
| Exploratory EFS parsimonious cause-specific Cox | Other regimen vs MAP | 0.099 | 0.381 | 162 | 80 | Schoenfeld-residual correlation with log event time |
| Exploratory EFS parsimonious cause-specific Cox | GLOBAL approximate |  | 0.366 | 162 | 80 | Approximate global aggregation of term-level tests |
| Exploratory pulmonary-metastasis parsimonious cause-specific Cox | Intermediate recovery phenotype vs favorable recovery | 0.110 | 0.488 | 147 | 42 | Schoenfeld-residual correlation with log event time |
| Exploratory pulmonary-metastasis parsimonious cause-specific Cox | Persistent recovery failure vs favorable recovery | -0.302 | 0.052 | 147 | 42 | Schoenfeld-residual correlation with log event time |
| Exploratory pulmonary-metastasis parsimonious cause-specific Cox | age | -0.092 | 0.562 | 147 | 42 | Schoenfeld-residual correlation with log event time |
| Exploratory pulmonary-metastasis parsimonious cause-specific Cox | Male vs female | -0.056 | 0.727 | 147 | 42 | Schoenfeld-residual correlation with log event time |
| Exploratory pulmonary-metastasis parsimonious cause-specific Cox | Tumor size (cm) | -0.062 | 0.695 | 147 | 42 | Schoenfeld-residual correlation with log event time |
| Exploratory pulmonary-metastasis parsimonious cause-specific Cox | AP vs MAP | -0.171 | 0.280 | 147 | 42 | Schoenfeld-residual correlation with log event time |
| Exploratory pulmonary-metastasis parsimonious cause-specific Cox | Other regimen vs MAP | 0.098 | 0.538 | 147 | 42 | Schoenfeld-residual correlation with log event time |
| Exploratory pulmonary-metastasis parsimonious cause-specific Cox | GLOBAL approximate |  | 0.491 | 147 | 42 | Approximate global aggregation of term-level tests |

Note: All event-related analyses are exploratory.

## Supplementary Table S9. Exploratory pulmonary-metastasis competing-risk analyses

**Panel A. Pulmonary-metastasis competing-event summary.**

| **recovery phenotype** | **N** | **pulmonary metastasis events** | **competing deaths before pulmonary metastasis** | **administrative or loss censoring** |
| --- | --- | --- | --- | --- |
| Overall | 147 | 42 | 15 | 90 |
| Favorable recovery | 60 | 7 | 6 | 47 |
| Intermediate recovery phenotype | 41 | 8 | 5 | 28 |
| Persistent recovery failure | 46 | 27 | 4 | 15 |

**Panel B. Fine-Gray pulmonary-metastasis model.**

| **term** | **estimate log subdistribution hazard** | **subdistribution HR** | **95% CI low** | **95% CI high** | **p value** | **converged** | **iterations** | **N** | **events of interest** | **competing events** | **analysis status** | **interpretive scope** |
| --- | --- | --- | --- | --- | --- | --- | --- | --- | --- | --- | --- | --- |
| Intermediate recovery phenotype vs favorable recovery | 0.511 | 1.666 | 0.600 | 4.629 | 0.327 | Yes | 6 | 147 | 42 | 15 | Exploratory | Competing-risk sensitivity analysis; no causal or validated prognostic claim |
| Persistent recovery failure vs favorable recovery | 1.872 | 6.501 | 2.762 | 15.302 | <0.001 | Yes | 6 | 147 | 42 | 15 | Exploratory | Competing-risk sensitivity analysis; no causal or validated prognostic claim |
| Age, per SD | 0.039 | 1.040 | 0.779 | 1.389 | 0.792 | Yes | 6 | 147 | 42 | 15 | Exploratory | Competing-risk sensitivity analysis; no causal or validated prognostic claim |
| Male vs female | 0.228 | 1.256 | 0.660 | 2.388 | 0.487 | Yes | 6 | 147 | 42 | 15 | Exploratory | Competing-risk sensitivity analysis; no causal or validated prognostic claim |
| Tumor size, per SD | 0.097 | 1.102 | 0.792 | 1.533 | 0.564 | Yes | 6 | 147 | 42 | 15 | Exploratory | Competing-risk sensitivity analysis; no causal or validated prognostic claim |
| AP vs MAP | 0.155 | 1.167 | 0.560 | 2.432 | 0.680 | Yes | 6 | 147 | 42 | 15 | Exploratory | Competing-risk sensitivity analysis; no causal or validated prognostic claim |
| Other regimen vs MAP | 0.043 | 1.044 | 0.482 | 2.264 | 0.913 | Yes | 6 | 147 | 42 | 15 | Exploratory | Competing-risk sensitivity analysis; no causal or validated prognostic claim |

## Supplementary Table S10. Robustness across pathological-response definitions

| **analysis** | **N** | **estimate type** | **term** | **estimate** | **CI low** | **CI high** | **p value** |
| --- | --- | --- | --- | --- | --- | --- | --- |
| Necrosis <80%: complete-case parsimonious Firth | 158 | OR | Intermediate recovery phenotype vs favorable recovery | 1.535 | 0.606 | 3.888 | 0.366 |
| Necrosis <80%: complete-case parsimonious Firth | 158 | OR | Persistent recovery failure vs favorable recovery | 3.853 | 1.621 | 9.156 | 0.002 |
| Necrosis <80%: complete-case fully adjusted Firth | 158 | OR | Intermediate recovery phenotype vs favorable recovery | 1.395 | 0.537 | 3.626 | 0.495 |
| Necrosis <80%: complete-case fully adjusted Firth | 158 | OR | Persistent recovery failure vs favorable recovery | 3.637 | 1.491 | 8.876 | 0.005 |
| Proportional-odds ordered logit for higher Huvos grade | 162 | common OR for higher response category | Persistent recovery failure vs favorable recovery | 0.208 | 0.101 | 0.426 | <0.001 |
| Binary cumulative logit Huvos > 1 | 162 | OR for higher response category | Persistent recovery failure vs favorable recovery | 0.200 | 0.076 | 0.527 | 0.001 |
| Binary cumulative logit Huvos > 2 | 162 | OR for higher response category | Persistent recovery failure vs favorable recovery | 0.157 | 0.065 | 0.376 | <0.001 |
| Binary cumulative logit Huvos > 3 | 162 | OR for higher response category | Persistent recovery failure vs favorable recovery | 0.277 | 0.053 | 1.436 | 0.126 |
| Robust OLS necrosis percentage | 158 | adjusted percentage-point difference persistent vs favorable | Persistent recovery failure vs favorable recovery | -10.265 | -15.723 | -4.808 | <0.001 |
| Median regression necrosis percentage | 158 | adjusted percentage-point difference persistent vs favorable | Persistent recovery failure vs favorable recovery | -11.539 | -17.115 | -5.964 | <0.001 |

Note: Percentage-based analyses include only patients with an exact necrosis percentage. Ordered Huvos and continuous necrosis analyses test robustness beyond the 90% binary threshold.

## Supplementary Table S11. Alternative recovery-phenotype definitions and T1/T2 incremental analysis

**Panel A. Alternative recovery-phenotype definitions.**

| **definition** | **favorable N** | **intermediate N** | **persistent N** | **reclassified vs primary rule** | **OR persistent vs favorable** | **95% CI low** | **95% CI high** | **p value** | **analysis role** |
| --- | --- | --- | --- | --- | --- | --- | --- | --- | --- |
| Primary T0-T3 rule | 65 | 45 | 52 | 0 | 5.717 | 2.416 | 13.526 | <0.001 | Primary |
| Protocol-aligned expanded persistent-failure rule | 65 | 30 | 67 | 15 | 4.089 | 1.913 | 8.741 | <0.001 | Sensitivity |
| T3-only median/upper-tertile categorization | 81 | 27 | 54 | 18 | 4.051 | 1.838 | 8.929 | <0.001 | Sensitivity |

**Panel B. Incremental value of the T1/T2-derived slope beyond the continuous T3 score.**

| **N with at least 3 timepoints** | **likelihood ratio chisq for slope given T3** | **degrees of freedom** | **p value** | **slope OR per 30 day unit** | **95% CI low** | **95% CI high** | **interpretation** |
| --- | --- | --- | --- | --- | --- | --- | --- |
| 157.000 | 1.989 | 1 | 0.158 | 2.255 | 0.716 | 7.103 | T1/T2-derived longitudinal slope did not provide statistically detectable incremental information beyond T3. |

## Supplementary Table S12. Full-pipeline bootstrap of classification and primary effect estimates

| **metric** | **valid replicates** | **median** | **mean** | **q025** | **q975** |
| --- | --- | --- | --- | --- | --- |
| OR | 1000 | 5.698 | 6.536 | 2.481 | 15.727 |
| RR | 1000 | 2.035 | 2.085 | 1.461 | 3.017 |
| RD | 1000 | 0.368 | 0.367 | 0.202 | 0.538 |
| Standardized risk: favorable recovery | 1000 | 0.380 | 0.381 | 0.272 | 0.492 |
| Standardized risk: persistent recovery failure | 1000 | 0.747 | 0.748 | 0.623 | 0.862 |
| Favorable recovery, N | 1000 | 66.000 | 65.980 | 58.000 | 74.000 |
| Intermediate recovery phenotype, N | 1000 | 44.000 | 44.138 | 36.000 | 51.000 |
| Persistent recovery failure, N | 1000 | 52.000 | 51.882 | 46.000 | 55.000 |

Note: Each bootstrap replicate re-estimated T0 standardization, scores, distributional thresholds, phenotype assignment, and the primary effect model.

## Supplementary Table S13. Internal calendar-time analyses of phenotype stratification and prediction-model transportability

| **Analysis block** | **Training/test split** | **Model** | **Training N** | **Test N** | **Test events** | **AUC** | **Brier** | **Calibration intercept** | **Calibration slope** | **Group agreement** | **Interaction P** |
| --- | --- | --- | --- | --- | --- | --- | --- | --- | --- | --- | --- |
| Clinical-variable multivariable prediction model | 2014-2019 training / 2020-2025 test | Recovery phenotype | 92 | 70 | 37 | 0.493 | 0.253 | 0.129 | -0.081 |  |  |
| Clinical-variable multivariable prediction model | 2014-2019 training / 2020-2025 test | Continuous T3 score | 92 | 70 | 37 | 0.609 | 0.240 | -0.044 | 1.064 |  |  |
| Clinical-variable multivariable prediction model | 2014-2020 training / 2021-2025 test | Recovery phenotype | 107 | 55 | 29 | 0.599 | 0.245 | -0.070 | 1.198 |  |  |
| Clinical-variable multivariable prediction model | 2014-2020 training / 2021-2025 test | Continuous T3 score | 107 | 55 | 29 | 0.642 | 0.236 | 0.035 | 1.200 |  |  |
| Clinical-variable multivariable prediction model | 2014-2021 training / 2022-2025 test | Recovery phenotype | 118 | 44 | 25 | 0.600 | 0.257 | 0.448 | 0.552 |  |  |
| Clinical-variable multivariable prediction model | 2014-2021 training / 2022-2025 test | Continuous T3 score | 118 | 44 | 25 | 0.592 | 0.253 | 0.413 | 0.632 |  |  |
| Phenotype or T3 score used alone | 2014-2019 / 2020-2025 | Recovery phenotype alone | 92 | 70 | 37 | 0.729 | 0.210 | -0.025 | 1.677 | 0.957 |  |
| Phenotype or T3 score used alone | 2014-2019 / 2020-2025 | Continuous T3 score alone | 92 | 70 | 37 | 0.699 | 0.220 | 0.029 | 0.870 |  |  |
| Phenotype or T3 score used alone | 2014-2020 / 2021-2025 | Recovery phenotype alone | 107 | 55 | 29 | 0.706 | 0.216 | 0.075 | 1.155 | 0.964 |  |
| Phenotype or T3 score used alone | 2014-2020 / 2021-2025 | Continuous T3 score alone | 107 | 55 | 29 | 0.666 | 0.234 | 0.140 | 0.615 |  |  |
| Phenotype or T3 score used alone | 2014-2021 / 2022-2025 | Recovery phenotype alone | 118 | 44 | 25 | 0.708 | 0.222 | 0.593 | 1.428 | 0.955 |  |
| Phenotype or T3 score used alone | 2014-2021 / 2022-2025 | Continuous T3 score alone | 118 | 44 | 25 | 0.661 | 0.241 | 0.453 | 0.723 |  |  |
| Phenotype-by-era interaction | late era = diagnosis 2020-2025 | Recovery phenotype x treatment era |  |  |  |  |  |  |  |  | 0.705 |

Note: These internal calendar-time analyses do not constitute external validation. Phenotype-only stratification and multivariable prediction transportability answer different questions.

## Supplementary Table S14. Primary-cohort inclusion, baseline selection audit, and inclusion-weighted association

| **Analysis block** | **Variable or estimate** | **Included** | **Excluded** | **SMD** | **P value** | **Estimate** | **95% CI** |
| --- | --- | --- | --- | --- | --- | --- | --- |
| Included versus excluded candidates | tumor size cm | 8.70 | 9.90 | -0.459 | 0.023 |  |  |
| Included versus excluded candidates | albumin baseline g L | 41.89 | 42.74 | -0.289 | 0.202 |  |  |
| Included versus excluded candidates | OS event | 14.8% | 23.1% | -0.212 | 0.384 |  |  |
| Included versus excluded candidates | ALP baseline U L | 192.38 | 177.62 | 0.199 | 0.300 |  |  |
| Included versus excluded candidates | baseline metastasis | 9.3% | 15.4% | -0.187 | 0.307 |  |  |
| Included versus excluded candidates | CRP baseline mg L | 7.47 | 8.23 | -0.120 | 0.502 |  |  |
| Included versus excluded candidates | infection any NAC | 19.1% | 15.4% | 0.099 | 0.790 |  |  |
| Included versus excluded candidates | dose reduction | 14.8% | 11.5% | 0.097 | 1.000 |  |  |
| Included versus excluded candidates | NAC cycles | 3.60 | 3.54 | 0.078 | 0.696 |  |  |
| Included versus excluded candidates | EFS event | 42.6% | 46.2% | -0.072 | 0.832 |  |  |
| Included versus excluded candidates | poor pathological response | 54.3% | 57.7% | -0.068 | 0.833 |  |  |
| Included versus excluded candidates | dose delay | 31.5% | 34.6% | -0.067 | 0.821 |  |  |
| Included versus excluded candidates | LDH baseline U L | 252.01 | 248.31 | 0.060 | 0.761 |  |  |
| Included versus excluded candidates | BMI | 21.68 | 21.79 | -0.035 | 0.874 |  |  |
| Included versus excluded candidates | age | 21.72 | 21.50 | 0.023 | 0.915 |  |  |
| Included versus excluded candidates | GCSF use any NAC | 50.6% | 50.0% | 0.012 | 1.000 |  |  |
| Included versus excluded candidates | sex | 53.7% | 53.8% | -0.003 | 1.000 |  |  |
| Complete-case inclusion weighting | Eligible/included/excluded N=188/162/26; weight ESS=160.6 |  |  |  |  | Adjusted RR 2.06 | 1.44-2.94 |

Note: SMDs describe baseline differences between included and excluded otherwise eligible candidates. Inclusion weighting addresses observed baseline predictors of complete T0-T3 availability but cannot remove selection related to unmeasured factors or conditioning on surgery.

# Supplementary Figures

## Supplementary Figure S1. Laboratory availability and primary-cohort inclusion.

Availability of laboratory components across treatment windows and flow into the T0-T3 primary cohort.

## Supplementary Figure S2. Correlation structure among score components.

Spearman correlations among transformed and standardized score components. The figure documents algebraic overlap and does not imply independent biological domains.

## Supplementary Figure S3. Score and classification sensitivity analyses.

Panel A shows adjusted odds ratios from leave-one-component, leave-one-domain, and nonredundant primitive-variable sensitivity analyses. Panel B shows paired internal-validation differences in AUC between the recovery phenotype and individual or combined simple biomarkers and continuous failure-score change; positive values favor the recovery phenotype, whereas confidence intervals crossing zero indicate no clear performance advantage.

## Supplementary Figure S4. Exploratory paired immune-subset findings.

Baseline-adjusted T3 immune-subset comparisons in 24 paired patients. Estimates are geometric mean ratios versus favorable recovery; multiplicity-adjusted results and selection-audit details are reported in Supplementary Table S6.

## Supplementary Figure S5. Chemotherapy-regimen distributions and heterogeneity.

Phenotype distributions and pathological-response rates by neoadjuvant regimen. No statistically detectable global phenotype-by-regimen interaction was observed, but regimen-specific estimates were imprecise.
